# A Longitudinal Study in Tunisia to Assess the Anti-RBD IgG and IgA Responses Induced by Three Different COVID-19 Vaccine Platforms

**DOI:** 10.3390/tropicalmed9030061

**Published:** 2024-03-13

**Authors:** Wafa Ben Hamouda, Mariem Hanachi, Sonia Ben Hamouda, Wafa Kammoun Rebai, Adel Gharbi, Amor Baccouche, Jihene Bettaieb, Oussema Souiai, Mohamed Ridha Barbouche, Koussay Dellagi, Melika Ben Ahmed, Chaouki Benabdessalem

**Affiliations:** 1 Laboratory of Transmission, Control and Immunobiology of Infections, Institut Pasteur de Tunis, Tunis El Manar University, Tunis 1002, Tunisia; wafabenhamoudaa@gmail.com (W.B.H.); soniabenhamoudaa@gmail.com (S.B.H.); adel.gharbi@pasteur.tn (A.G.); amorbaccouche65@gmail.com (A.B.); bettaiebjihene@yahoo.fr (J.B.); mohamedrb@agu.edu.bh (M.R.B.); melika.benahmed@pasteur.tn (M.B.A.); 2 Department of Biological Sciences, Faculty of Sciences of Tunis, Tunis El Manar University, Tunis 1068, Tunisia; 3 Laboratory of Bioinformatics, Biomathematics and Biostatistics LR16IPT09, Institut Pasteur de Tunis, Tunis El Manar University, Tunis 1002, Tunisia; mariem.hanachi@pasteur.utm.tn (M.H.); oussama.souiai@istmt.utm.tn (O.S.); 4 Laboratory of Medical Parasitology, Biotechnologies and Biomolecules, Institut Pasteur de Tunis, Tunis El Manar University, Tunis 1002, Tunisia; wafa.kammoun@pasteur.tn; 5 Faculty of Medicine of Tunis, Tunis El Manar University, Tunis 1068, Tunisia; 6 Department of Microbiology, Immunology, and Infectious Diseases, College of Medicine and Medical Sciences, Arabian Gulf University, Manama 329, Bahrain; 7 Pasteur Network, Institut Pasteur, 75724 Paris, France; koussay.dellagi@pasteur.fr

**Keywords:** COVID-19, RNA vaccines, inactivated vaccines, viral vector vaccines, IgG, IgA, kinetics

## Abstract

Background: Vaccination constitutes the best strategy against COVID-19. In Tunisia, seven vaccines standing for the three main platforms, namely RNA, viral vector, and inactivated vaccines, have been used to vaccinate the population at a large scale. This study aimed to assess, in our setting, the kinetics of vaccine-induced anti-RBD IgG and IgA antibody responses. Methods: Using in-house developed and validated ELISA assays, we measured anti-RBD IgG and IgA serum antibodies in 186 vaccinated workers at the Institut Pasteur de Tunis over 12 months. Results: We showed that RNA vaccines were the most immunogenic vaccines, as compared to alum-adjuvanted inactivated and viral-vector vaccines, either in SARS-CoV-2-naïve or in SARS-CoV-2-experienced individuals. In addition to the IgG antibodies, the vaccination elicited RBD-specific IgAs. Vaccinated individuals with prior SARS-CoV-2 infection exhibited more robust IgG and IgA antibody responses, as compared to SARS-CoV-2-naïve individuals. Conclusions: After following up for 12 months post-immunization, we concluded that the hierarchy between the platforms for anti-RBD antibody-titer dynamics was RNA vaccines, followed by viral-vector and alum-adjuvanted inactivated vaccines.

## 1. Introduction

In late 2019, the severe acute respiratory syndrome coronavirus 2 (SARS-CoV-2) emerged as the causative agent of coronavirus disease 2019 (COVID-19), leading to an unprecedented global pandemic. Several studies have shown that the COVID-19 severity and outcomes were determined by host immunity [1,2,3,4]. Vaccination is one of the most important strategies for accelerating viral clearance and protect against severe disease.

The receptor-binding domain (RBD) of the spike (S) protein binds the virus to the host cells through the receptor angiotensin-converting enzyme 2 (ACE2), mediating virus entry and triggering the host immune responses [5,6]. Consequently, the S-protein, more precisely its RBD, became a primary target for antibody-based immunotherapies and vaccines. While SARS-CoV-2 infection elicits the production of a large array of antibodies that recognize multiple viral proteins including structural and non-structural proteins, mainly antibodies to the spike protein have the potential to mediate protective immunity as they exhibit potent neutralizing activity [7,8,9].

To date, the World Health Organization (WHO) has granted “emergency use listing” for 13 vaccines against COVID-19. Several platforms have been used, namely the following: (i). For the first time, mRNA-based vaccines using modified sequences of the spike protein gene; (ii). DNA-based vaccines; (iii). Protein subunit vaccines; (iv). Inactivated virus vaccines; and (v). Recombinant viral vectors [10]. Among the 13 vaccines, only 4 have received authorization from the U.S. Food and Drug Administration (Pfizer-BioNTech (Pfizer Inc. and BioNTech SE, New York, NY, USA/Mainz, Germany), Moderna Moderna, Inc., Massachusetts, MA, USA), Janssen/Johnson & Johnson (Janssen Pharmaceuticals, Beerse/New Jersey, NJ, Belgium/USA), and Novavax (Novavax, Inc., Maryland, MD, USA) [11]. Even though Sputnik V, an adenoviral virus, had been developed early, after the start of the pandemic, and was the first COVID-19 vaccine to be deployed, it was only licensed in a few countries [12].

In Tunisia, a country of 12.5 million inhabitants, seven vaccines, namely BNT162b2, mRNA-1273, AZD1222/ChAdOx1, AD26.COV2. S, BBIBP-CorV, CoronaVac, and Sputnik V, have been widely used. According to the WHO data released in June 2023, more than 13.253 million doses of vaccines have been delivered in the country [13].

In order to understand how the vaccines behave in populations, it is important to monitor the development and the duration of virus-specific antibodies after COVID-19 vaccination in various endemic settings. By monitoring the kinetics of the post-vaccine antibody response, one can assess the potency of the vaccine [14].

Typically, vaccines administered either intramuscularly or intradermally have generated robust immunoglobulin (Ig) M and IgG responses. In some cases, the parenteral administration of vaccines had the potential to induce the release of secretory IgA [15]. There was compelling evidence supporting the protective role of IgA against SARS-CoV-2 infection [16,17,18,19]. Interestingly, the levels of IgA antibodies in the serum may have served as a valuable biomarker, as its correlated with the neutralizing activity of secretory IgA at the mucosal surface in infected individuals [20].

Studies on antibody responses induced by vaccination against COVID-19 have focused mainly on IgG [21,22,23,24,25,26,27,28,29] and less frequently on IgA dynamics [24,25,26,27,28,29]. Most of the studies investigating the systemic IgA response were associated with the use of RNA vaccines [24,25,27,28]. To the best of our knowledge, ours was the first study that comparatively examined the anti-RBD IgA response induced by three distinct platforms.

To this end, a prospective open-cohort of workers at the Institut Pasteur de Tunis, who had been vaccinated against SARS-CoV-2, participated in follow-up testing for the 12 months after vaccination using previously developed and validated serological tests for serum IgG and IgA antibodies against the RBD domain of SARS-CoV-2.

## 2. Materials and Methods

### 2.1. Study Participants

Between June 2021 and August 2022, we prospectively collected 871 samples from 186 Tunisian workers (>18 years) at the Institut Pasteur de Tunis who received SARS-CoV-2 vaccines from either Pfizer-BioNTech (BNT162b2), Moderna (mRNA-1273), Oxford-AstraZeneca (AZD1222, London, UK), Janssen (Ad26.CoV.S), Gamaleya (Sputnik V, Moscow, Russia), Sinovac (CoronaVac, Beijing, China), or Sinopharm (WIBP-CorV, Beijing, China), which were representative of the three main vaccine platforms used for the prevention of COVID-19, mRNA (Pfizer and Moderna, *n* = 133), viral vector (Oxford-AstraZeneca, Janssen, and Gamaleya, *n* = 31), and alum-adjuvanted inactivated viruses (Sinovac and Sinopharm, *n* = 22). All subjects participating in the study provided informed consent. The study protocol was approved by the Institut Pasteur de Tunis ethical committee (2021/34/I/V1).

Included subjects were interviewed face to face by trained interviewers to fill out a paper questionnaire that was used to identify the history of infection and co-morbidities. Sera samples were collected at 6 time-points, ranging from the baseline before vaccination to 12-months post-vaccination.

In order to detect SARS-CoV-2 exposure in the study participants, we combined two sources of information: the positive results of RT-PCR testing for SARS-CoV-2 [30] and/or detection of antibodies to the N protein by in-house ELISA test [31], prior to inclusion in the cohort or during the post-vaccination follow-up. Of note, for inactivated vaccine recipients, only positive PCRs for SARS-CoV-2 were considered. The PCR testing was carried out in a laboratory of clinical virology for routine testing of the workers of the Pasteur Institute of Tunis with suspected SARS-CoV-2 infection.

The cohort was categorized into two groups: individuals with a history of infection prior to vaccination (*n* = 93) and individuals naïve to infection (*n* = 93).

Detection of anti-N antibodies during the post-vaccination follow-up in participants who received the RNA or viral vector vaccines was indicative of a breakthrough infection, as these vaccines did not have or produce N antigen. As the presence of the antigen N was present in the inactivated vaccines, whereas it was not found in the other vaccines, this marker could not be used as a proxy of SARS-CoV-2 infection in recipients of inactivated vaccines. Sera sampled after a breakthrough SARS-CoV-2 infection were excluded from the study.

The vaccination protocol for all the 186 participants included the injection of two doses of vaccine with a 3–4-week interval between doses, except for individuals receiving the Janssen vaccine, who only required a single dose (*n* = 11). Moreover, during the follow-up, 82 participants received supplementary booster doses of vaccine and were, therefore, retrospectively excluded from the analysis.

### 2.2. Detection of Serum SARS-CoV-2 S-RBD-Specific IgG

We optimized the entire upstream and downstream process for the production of the RBD spike S1 protein of SARS-CoV-2 (Wuhan-hu-1 strain) using Sf9 insect cells/baculovirus vector expression system (BEVS) in order to develop an ELISA assay [32]. The developed ELISA to detect S-RBD-specific IgG antibodies was validated and cross-validated in various endemic African settings [31]. Briefly, serum samples were diluted at 1:400 in PBS-0.01% Tween 20 (PBS-T), and then incubated in ELISA plates (Maxisorp Nunc, Thermo Fisher Scientific, Massachusetts, MA, USA) coated with recombinant SARS-CoV-2 S–RBD protein (2 µg/mL, 50 µL/well). Positive and negative controls (corresponding to a pre-pandemic serum and a serum from a COVID-19 patient, respectively) and a threshold serum were included on each plate. The threshold serum corresponded to a pre-pandemic serum with an optical density (OD) corresponding precisely to the pre-determined cut-off (the 95th percentile) [32].

After incubation, wells were washed, and the peroxidase-labeled anti-IgG antibody (1:8000, Sigma-Aldrich, Merck KGaA, Darmstadt, Germany, A6029) was added. The substrate solution (TMB, BD Biosciences, Becton, Dickinson and Company, NJ, USA) was then added, and the samples were incubated for 10 min in the dark. After adding the stop solution (sulfuric acid (2N)), OD values were measured at 450 nm and 630 nm (Spectrophotometer Multiskan Go 1510 Sky, ThermoFisher Scientific, Vantaa, Finland). Finally, the arbitrary unit (AU) was calculated based on a ratio of OD values obtained from the samples and the threshold serum. A relative OD of ≥1 was considered positive.

### 2.3. Detection of Serum SARS-CoV-2 S-RBD-Specific IgA

The ELISA plates were coated overnight at 4 °C with 1µg/mL of recombinant S-RBD protein (50 µL/well). After blocking with 4% bovine serum albumin in PBS-T for 1 h at 37 °C, sera samples were added at 1:100 dilution with PBS-T, and incubated at 37 °C for 2 h. After washing six times, peroxidase-labeled anti-IgA (Sigma-Aldrich, A0295) diluted at 1:8000 in PBS-T was added and then incubated at 37 °C for 1 h. After six washes, 50 µL of 3,3′,5,5′-Tetramethylbenzidine TMB was added. Plates were incubated for 10 min and then stopped by adding 50 µL of sulfuric acid (2N), and the optical density was measured at 450/630 nm.

### 2.4. Data Visualization and Statistical Analysis

Statistical analyses were performed using GraphPad Prism 8.4.3 and R version 4.3.1. Significant differences in multiple comparisons were analyzed by Kruskal–Wallis test. Moreover, the Mann–Whitney test (non-normal distribution) or unpaired *t*-test (normal distribution) were assessed to compare the two groups. Spearman’s correlations and scatterplots were estimated and visualized using ggpubr R package (v0.6).

The association of breakthrough infections, prior SARS-CoV-2 infection, and vaccine platforms were assessed with the Chi-squared test.

## 3. Results

### 3.1. Study Population

A total of 186 individuals were included in this study. All of them were workers at the Institut Pasteur de Tunis. The participants received two doses of vaccines at 34 ± 11 days interval of the following vaccines: Pfizer, Moderna, AstraZeneca, Gamaleya, Sinovac, or Sinopharm.

The ages of participants ranged from 23 to 71 (median = 45). A total of 39% were male, and 61% were female, yielding an M/F sex ratio of 0.63. The most frequent co-morbidities were arterial hypertension and obesity (11.29% for each), followed by diabetes (6.45%) and cardiovascular diseases (2.69%).

**Figure 1:**
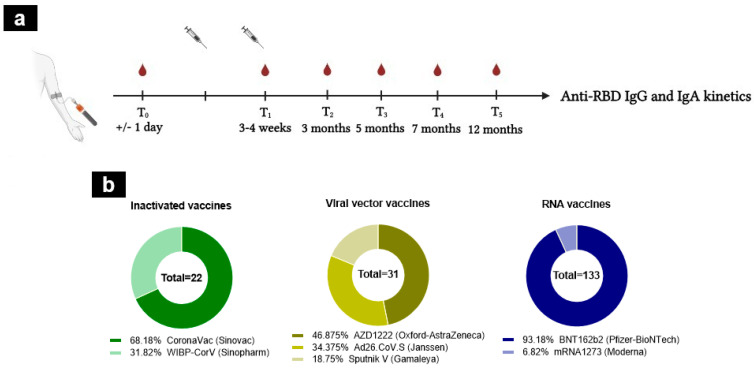
Study design and vaccine regimens. (**a**) The design of the cohort study. A total of 871 samples were collected during the longitudinal follow-up of 12 months, from individuals naïve to SARS-CoV-2 infection. (**b**) The cohort study was divided into three groups according to vaccine platforms: inactivated (*n* = 22, CoronaVac (dark green), WIBP-CorV (light green)), RNA (*n* = 133, BNT162b2 (dark blue), mRNA1273 (light blue)), and viral vector vaccines (*n* = 31, AZD1222 (dark yellow), Ad26.CoV.S (yellow), Sputnik V (light yellow)). The number inside the circle shows the total number.

A total of 871 samples were collected over 6 follow-up visits; pre-vaccination visits (T0); at 3–4 weeks after the first immunization (corresponding to 2–5 days after the second dose, except for individuals receiving the Janssen vaccine) (T1), at 3 months (T2), at 5 months (T3), at 7 months (T4), and at 12 months (T5) (Figure 1a). The samples were grouped according to the type of vaccine received: RNA (Pfizer and Moderna), viral vector (AstraZeneca, Janssen, and Gamaleya), and alum-adjuvanted inactivated vaccines (Sinovac and Sinopharm) (Figure 1b). No significant differences were noted between groups in terms of age (*p* > 0.05).

Sera from individuals who were infected with SARS-CoV-2 during the follow-up or had received an additional booster vaccine dose were excluded from the study.

Based on the occurrence (or not) of an infection by SARS-CoV-2 prior to the vaccine administration, our investigation showed that 93 participants (50%) were SARS-CoV-2-experienced versus 93 participants (50%) who were naïve to the virus. During the follow-up, 82 individuals received a homologous or heterologous booster vaccine (*n* = 70 or *n* = 14 respectively), as described in Table 1. The 14 individuals who received a heterologous booster dose included 10 SARS-CoV-2-naïve individuals and 4 SARS-CoV-2-experienced individuals. Among them, four individuals who had initially received viral vector vaccines and eight individuals who had received inactivated vaccines received a Pfizer booster vaccine. Additionally, one individual who had received inactivated vaccines received a Janssen booster vaccine, and another individual who had initially received an RNA vaccine (Pfizer) received a Moderna booster vaccine. The sera collected after the booster (the third dose, either homologous or heterologous) were excluded from the analysis.

During the follow-up, breakthrough infections after primary vaccination (without booster) occurred after vaccination in 46 (49.46%) SARS-CoV-2-naïve participants and in 35 (37.63%) participants who had a history of SARS-CoV-2 infection before vaccination (*p* = 0.1039) (Table 2). Interestingly, the rates of breakthrough infections were variable within the different vaccine groups, with the highest percentage in inactivated vaccine group. When we compared the rate of breakthrough infections using the Chi-squared test and considering the three types of vaccines between the SARS-CoV-2-naïve and -experienced groups, only the inactivated-vaccine recipients showed a significant difference. Accordingly, in this group, SARS-CoV-2-naïve individuals had a higher rate of breakthrough infection, as compared to the SARS-CoV-2-experienced individuals (*p* = 0.0133) (Table 2).

Considering that the group of individuals who had received the RNA vaccines was the largest, we grouped together individuals who received inactivated vaccines with those who had viral vector vaccines (designated hereafter as the non-RNA vaccine group). In the group of SARS-CoV-2-naïve vaccine recipients, the rate of breakthrough infection during follow-up was statistically comparable between the RNA and the non-RNA vaccine groups (*p* = 0.6029), using a Chi-squared test. In contrast, in the group of vaccinees with prior SARS-CoV-2 infection, the rate of breakthrough infections during follow-up was significantly higher in the group of RNA vaccine recipients, as compared to the group of non-RNA vaccine recipients (*p* = 0.0067). We observed that in the RNA vaccine group that most breakthrough infections had occurred rather late during the follow-up (T4 and T5) (Table 2).

### 3.2. Anti-RBD IgG Kinetics Elicited by Different Vaccine Platforms in SARS-CoV-2-Naïve Individuals and in Those with Pre-Vaccination History of SARS-CoV-2 Infection

**Figure 2:**
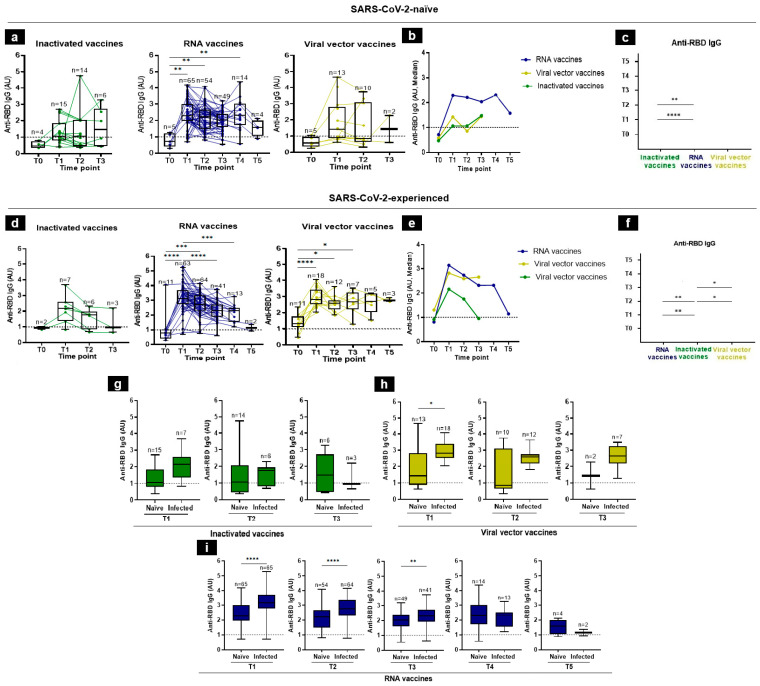
Anti-RBD IgG responses following vaccination in SARS-CoV-2-naïve and -experienced individuals. (**a**–**f**) RBD-specific IgG antibody kinetics, after vaccination, were assessed by an in-house ELISA test at 6 time-points from the baseline (T0), 3–4 weeks after the first immunization (T1), 3 months (T2), 5 months (T3), 7 months (T4), to 12 months (T5), in individuals who, prior to vaccine administration, were either naïve to SARS-CoV-2 (**a**) or were infected with SARS-CoV-2 (**d**). (**b**,**e**) Summary plots show anti-RBD IgG median levels in SARS-CoV-2- naïve (**b**) and -experienced (**e**) individuals. (**c**,**f**) Side-by-side comparison of IgG antibody levels between platforms in SARS-CoV-2-naïve (**c**) and -experienced (**f**) individuals. (**g**–**i**) Comparison of anti-RBD IgG levels in SARS-CoV-2-naïve and -experienced groups at each time-point elicited by inactivated (**g**), viral vector (**h**), and RNA (**i**) vaccines. Antibodies induced by inactivated, RNA, and viral vector vaccines were shown in green, blue, and yellow, respectively. Individual subjects are shown as connecting lines. The anti-RBD IgG cut-off was 1 AU (dashed line). Each dot stands for an individual, and error bars are the mean value and standard deviation of the distribution. The differences of anti-RBD IgG AU between vaccine platforms were calculated using the Kruskal–Wallis test, Mann–Whitney, or unpaired *t*-test (* *p* < 0.05, ** *p* < 0.01, *** *p* < 0.001, and **** *p* < 0.0001).

We first assessed the kinetics of anti-RBD IgG antibodies at all included time-points. In SARS-CoV-2-naïve participants, the levels of IgG antibodies specific to the RBD protein were almost undetectable at baseline, considering all platforms (Figure 2a,b). For RNA vaccines, Figure 2b shows that the median IgG levels increased rapidly at T1, remained high until T4, and then declined at T5 (median levels were at T1 = 2.2881, T2 = 2.20895, T3 = 2.034, T4 = 2.301, and T5 = 1.57665 AU). For inactivated and viral vector vaccines, the median IgG levels increased progressively until T3 (Figure 2b). Indeed, the median of the anti-RBD IgGs in response to inactivated vaccines was at T1 = 1.0559, at T2 = 1.0565, and at T3 = 1.46225 AU (Figure 2a,b). This increase should be taken with caution since the number of individuals followed-up at T4 (without breakthrough infection or boosted) was very low (*n* = 2). In response to the viral vector vaccines, the medians were 1.4239, 0.8569, and 1.4422 AU, at T1, T2, and T3, respectively (Figure 2b). It is worth noting that we lost long-term kinetics during the follow-up period on the recipients of inactivated vaccines and viral vector vaccines (at T4 and T5) that received a booster or experienced breakthrough infections. Overall, the RNA vaccines yielded the highest anti-RBD IgG response at early time-points (Figure 2b). Consistently, the side-by-side comparisons of IgG antibody levels induced by diverse vaccine platforms showed significantly higher anti-RBD IgG levels for the RNA vaccine group than in the inactivated one, at both T1 and T2 (Figure 2c).

In vaccine recipients who had a history of SARS-CoV-2 infection prior to vaccine administration, the IgG kinetics were almost different from those of the SARS-CoV-2-naïve group: the IgG antibody levels peaked rapidly at T1, considering all three platforms, while in the naïve group, it peaked only at T1 in RNA-vaccine group (Figure 2d,e). In the RNA vaccine group, for example, the IgG median levels were 0.8159 at T0, 3.1431 at T1, 2.73415 at T2, 2.3174 at T3, 2.3213 at T4, and 1.14975 AU at T5 (Figure 2d,e). In side-by-side comparisons, we showed that the RBD-specific IgG titers induced by the RNA vaccines were significantly higher than the inactivated vaccines at T1 and T2. Of note, the viral vector vaccines induced higher antibody titers than the inactivated vaccines at T2 and T3. No significant difference was observed between the RNA- and viral-vector-vaccine groups (Figure 2f).

In general, and as expected, the post-vaccination IgG response was higher in previously SARS-CoV-2-experienced individuals than in SARS-CoV-2-naïve individuals, and the difference was significant in response to viral vector vaccines at T1 and in response to RNA vaccines at T1, T2, and T3 (Figure 2g–i).

### 3.3. Anti-RBD IgA Kinetics Elicited by Different Vaccine Platforms in SARS-CoV-2-Naïve Individuals and in Those with Pre-Vaccination History of SARS-CoV-2 Infection

To assess the anti-RBD IgA kinetics in response to COVID-19 vaccination, we developed and validated an in-house ELISA test to detect the IgA isotype directed against the RBD antigen (Figure S1).

**Figure 3:**
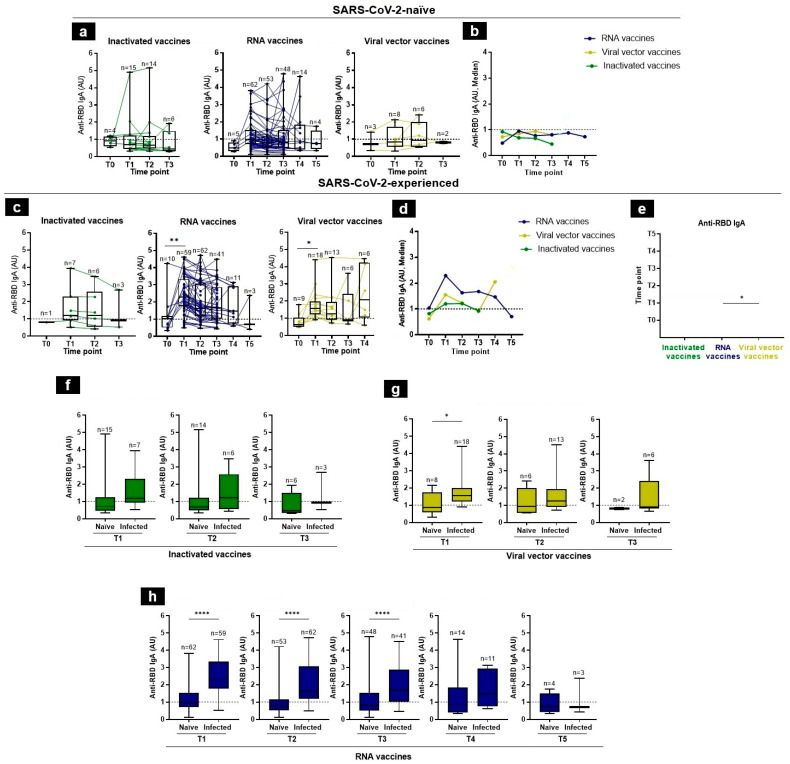
Anti-RBD IgA responses following vaccination in SARS-CoV-2-naïve and -experienced individuals. (**a**,**c**) RBD-specific IgA antibody kinetics, after vaccination, were assessed by an in-house ELISA test at 6 time-points, from baseline (T0), 3–4 weeks after the first immunization (T1), 3 months (T2), 5 months (T3), 7 months (T4), to 12 months (T5), in SARS-CoV-2-naïve (**a**) and -experienced (**c**) individuals. (**b**,**d**) Summary plots show anti-RBD IgA median levels in SARS-CoV-2-naïve (**b**) and -experienced (**d**) individuals. (**e**) Side-by-side comparison of IgA antibody levels between platforms in SARS-CoV-2-experienced. (**f**–**h**) Comparison of anti-RBD IgA levels in SARS-CoV-2-naïve and -experienced groups at each time-point, elicited by inactivated (**f**), viral vector (**g**), and RNA (**h**) vaccines. Antibodies induced by inactivated, RNA, and viral vector vaccines are shown with green, blue, and yellow, respectively. Individual subjects are shown as connecting lines. The anti-RBD IgA cut-off was 1 AU (dashed line). Each dot stands for an individual, and error bars are the mean value and standard deviation of the distribution. Statistics were calculated using the Kruskal–Wallis test, Mann–Whitney, or unpaired *t*-test (* *p* < 0.05, ** *p* < 0.01, and **** *p* < 0.0001).

In SARS-CoV-2-naïve individuals, the induction of RBD-specific IgA response was highly variable among different individuals in the same group (Figure 3a). IgA anti-RBD antibodies were detectable in 50%, 26.67%, and 28.57%, at T1, T2, and T3, respectively, in the group of inactivated vaccine recipients (Figure 3a), while in the mRNA vaccine platform, IgA was present in 38.71% at T1, 26.41% at T2, 29.17% at T3, 42.86% at T4, and 25% at T5 (Figure 3a). IgA had 33.33%, 25%, and 50% detection rates for the viral vector recipients at T0, T1, and T2, respectively (Figure 3a). Overall, our data showed that the median levels of the IgA antibodies were very low among the SARS-CoV-2-naïve individuals among the different groups of vaccine recipients (Figure 3b).

In contrast, higher levels of IgA antibodies were found in vaccine recipients with a prior history of SARS-CoV-2 infection and was similar among the different vaccine groups. However, the IgA levels were lower than the IgG antibodies. Indeed, the median anti-RBD IgA levels in response to the inactivated vaccines were T1 = 1.1898, T2 = 1.19845, and T3 = 0.9216 AU. For the RNA vaccines, the median IgA levels increased rapidly at T1 remained stable between T3 and T4, and then declined at T5 (at T1 = 2.2909, T2 = 1.62375, T3 = 1.6744, T4 = 1.4639, and T5 = 0.7022 AU). Those responding to the viral vector vaccines were 1.5581, 1.2444, 0.9162, and 2.0565 AU, at T1, T2, T3, and T4, respectively (Figure 3c,d). The IgA RBD-specific titers were significantly higher in response to RNA vaccines than those observed for viral vector vaccines at T1 (Figure 3e). One month after vaccination, there was a decline in specific RBD IgA levels in all groups, except in viral vector recipients who showed an increase in T4, which might not be informative due to the small available samples tested (*n* = 6) (Figure 3d,e).

Finally, the IgA post-vaccination response was significantly higher in SARS-CoV-2-experienced individuals, as compared to SARS-CoV-2-naïve individuals (Figure 3f–h). These observations illustrated the synergistic effect of pre-vaccination infection and vaccination to induce a good IgA response.

### 3.4. Association between Vaccine-Elicited IgG and IgA Responses

**Figure 4:**
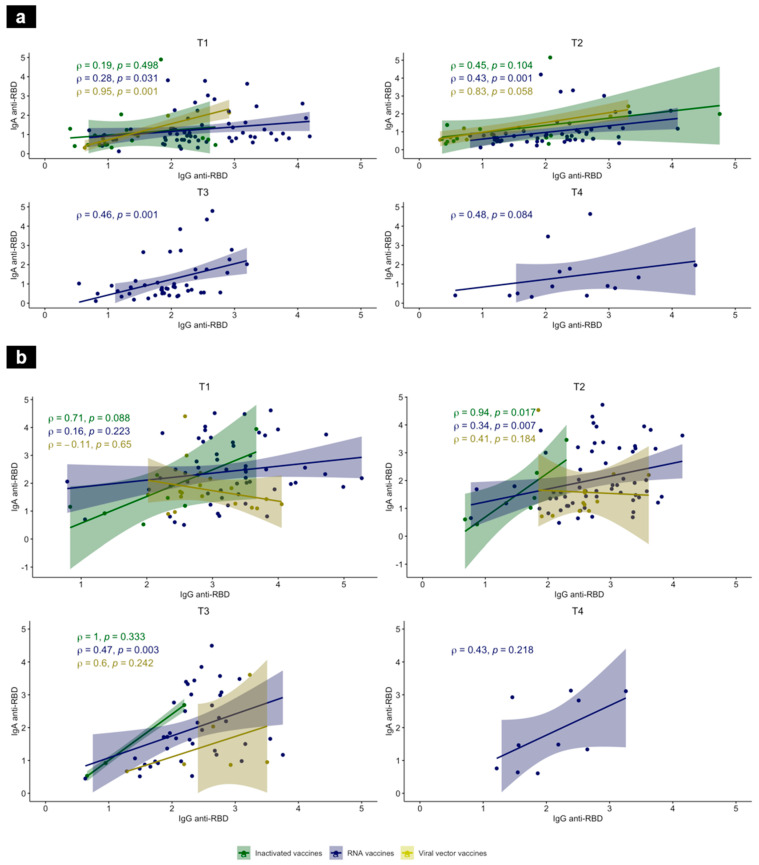
Correlations between anti-RBD IgG and IgA in SARS-CoV-2-naïve (**a**) and -experienced individuals (**b**) at T1, T2, T3, and T4, after vaccination. Scatterplot features a linear regression line, fitted to the data and a shaded band representing the 95% confidence interval. Anti-RBD IgA (AU, *Y*-axis) versus anti-RBD IgG (AU, *X*-axis), 3–4 weeks after the first immunization, and then 3 months, 5 months, and 7 months, using inactivated (green), RNA (blue) and viral vector (yellow) vaccines. Associations were calculated using a nonparametric Spearman’s correlation with a 95% confidence interval and a two-tailed *p*-value < 0.05 was statistically significant. ρ: Spearman’s correlation coefficient.

The correlation between the IgG and IgA anti-RBD antibodies was assessed at each time-point and for each vaccine platform. Our data showed a correlation in both previously SARS-CoV-2-experienced and -naïve individuals. In the naïve group, a strong correlation was found in the viral vector group at T1 (Rho = 0.95 with *p* = 0.001) between RBD-specific IgG and IgA RBD-specific IgG and IgA. In the RNA vaccine group, this correlation was moderate at T1, T2, and T3 (Figure 4a). In the SARS-CoV-2-experienced group, this correlation between the RBD-specific IgG and IgA was similarly moderate in the RNA vaccine group at T2 and T3 (Rho = 0.34 and 0.47 with *p* = 0.007 and *p* = 0.003, respectively). Even though in the inactivated vaccine group at T2 the correlation was strong between the anti-RBD IgG and IgA (Rho = 0.94, *p* = 0.017), the small number of tested subjects did not allow for a more robust conclusion (Figure 4b).

### 3.5. The Effect of Age on Antibody Response over Time in Vaccinated Individuals

No significant correlations were detected between antibody titers and age in different vaccine platforms, except among the viral-vector, SARS-CoV-2-experienced recipients, where a moderate positive association between IgG levels and age was observed at two time-points, T1 and T2 (Rho = 0.5661 and 0.6678 respectively) (Figure S2).

**Figure 5:**
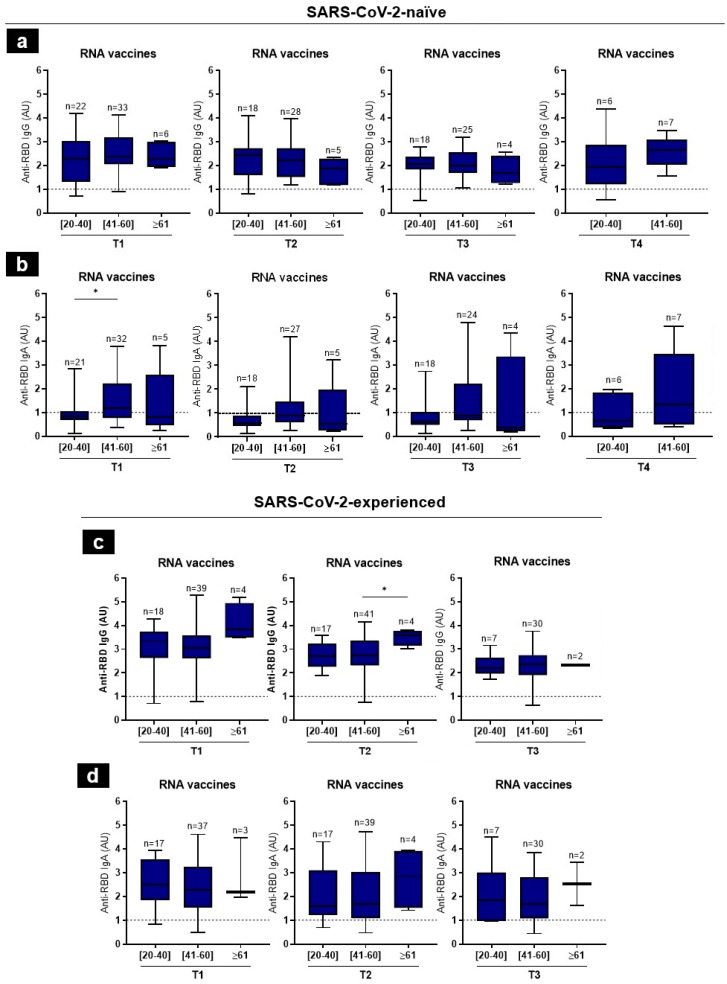
RBD-specific IgG and IgA antibodies in vaccinated individuals, stratified by age, over 12 months. Comparison of antibody levels elicited by RNA vaccines between three age groups in naïve (**a**,**b**) and SARS-CoV-2-experienced (**c**,**d**) individuals, at each time-point. The anti-RBD antibody cut-off was 1 AU (dashed line). Error bars stand for the mean value and standard deviation of the distribution. Statistics were calculated using the Kruskal–Wallis test (* *p* < 0.05).

We arbitrarily categorized the recipients of the RNA vaccines into three groups: 20–40 years old; 41–60 years old; and 60 years old and older. There were few differences in the anti-RBD IgG and IgA levels among the age groups of the SARS-CoV-2-naïve and -experienced vaccine recipients (Figure 5). Consistently, the anti-RBD IgA levels in the SARS-CoV-2-naïve group were significantly higher in the 41–60-years-old group, as compared to the 20–40-years-old group, at T1. Moreover, the eldest group (over 60 years) had significantly higher levels of anti-RBD IgG than the younger groups, at T2. This could be explained by the very small number of individuals (*n* = 4) in the subgroup (Figure 5b,c).

## 4. Discussion

The immune response elicited by vaccination involves multiple players, including innate, humoral, cellular, and cytokine responses. Although the humoral immune response is merely a part of the broader immune response, it has stood out as more easily detectable than other components due to its widespread use and standardized assessment methods [33,34]. Detecting the antibody response to SARS-CoV-2 has served multiple purposes, such as identifying past infections, diagnosing current infections, and assessing the efficacy of vaccines [35]. The role of antibodies, including neutralizing and non-neutralizing, as a correlate of protection for vaccines against numerous viral diseases has been established [36,37].

In the present study, we assessed the IgG and IgA responses to RBD after SARS-CoV-2 vaccination in a longitudinal open-cohort study of 186 individuals, with or without a prior history of SARS-CoV-2 infection. In Tunisia, the national vaccination strategy had initially targeted high-risk groups, namely people aged 60 years old and over; healthcare workers; and patients with chronic diseases. Consequently, the present study was conducted on a selected population of workers at the Institut Pasteur de Tunis who were eligible for the rapid vaccination program. The participants involved were mostly of a younger age, predominantly female, and had either not been infected (no symptoms) or mild manifestations of COVID-19 during prior infections. A recent study reported that vaccines from the same platforms had elicited similar rates of specific anti-S antibodies [23,38]. Moreover, the numbers in the different vaccine groups had been low except in the RNA vaccine group. Thus, the vaccine recipients in our study were categorized into three groups based on the type of vaccine platform. The first group received Pfizer or Moderna (RNA vaccine); the second group received AstraZeneca, Janssen, or Gamaleya (viral vector vaccine); and the latter group received Sinovac or Sinopharm (alum-adjuvanted inactivated vaccine).

The findings of our study agreed with the results from numerous studies worldwide reporting that anti-RBD IgG had reached maximum levels 3–4 weeks after immunization [23,24,28,39,40]. Moreover, consistent with a previous report [23], the recipients of the RNA vaccines (Pfizer and Moderna) had higher peak antibody titers than the viral-vector and alum-adjuvanted inactivated-vaccine recipients. In line with previous studies, our results showed that after the initial peak, the IgGs antibodies dropped to lower levels, quite quickly, for up to 3 months, for inactivated and viral vector vaccines [22,40,41], and more progressively for RNA vaccines, at up to 7 months after immunization [22,24,28].

Although the numbers of the samples in each group were not matched, our side-by-side comparisons revealed that the RNA vaccines had yielded the highest levels of IgG anti-RBD antibodies, as compared to alum-adjuvanted inactivated and viral vector vaccines, in both the SARS-CoV-2-naïve and the SARS-CoV-2-experienced groups. These findings were consistent with previous reports demonstrating that the mRNA vaccines had elicited higher IgG titers and greater antibody affinity, as compared to other types of vaccines, such as viral vector vaccines (ChAdOx1-S, Sputnik V, Ad26.COV2.S) and inactivated vaccines (BBIBP-CorV) [22,23,38,42]. Interestingly, our data also showed that the viral vector vaccines had induced higher antibody titers than the alum-adjuvanted inactivated vaccines, similar to previously reported results [35,38,40].

Several studies demonstrated that IgA exhibited more potent antiviral properties, as compared to IgG, for influenza and SARS-CoV-2 [17,43,44,45]. Interestingly, it was reported that the levels of systemic IgA response following mRNA vaccination had been associated with protection, as reflected in the risk of subsequent breakthrough infection [25]. Moreover, it was demonstrated that a full BNT162b2 vaccination had induced robust systemic IgG and IgA responses directed towards the S-protein and the RBD in serum samples. However, there were limited IgG and IgA antibodies in saliva [46].

Our results showed that the level of the IgA response differed from that of the IgG response. Consistent with published data [27], the COVID-19 mRNA vaccine (Moderna and Pfizer) elicited the production of spike-specific IgA, showing similar patterns of induction and the time to reach peak levels than spike-specific IgG, but a more rapid decline in serum levels following both the first and second doses of the vaccine [27]. In our study, the median level of the RBD-specific IgA antibodies in SARS-CoV-2-naïve individuals was undetectable, except in the alum-adjuvanted inactivated-vaccine group seven months after immunization, which was the median of only two patients, with high discrepancy. In contrast, the kinetics of the IgA antibodies in SARS-CoV-2-experienced recipients were similar to those observed for the IgG antibodies, although at a lower level. Similar results reported that mRNA vaccination had induced a weak IgA response in SARS-CoV-2-naïve individuals, but a stronger one in SARS-CoV-2-experienced individuals [47].

As expected, we found that the post-vaccination IgG and IgA responses were higher in SARS-CoV-2-experienced individuals than in SARS-CoV-2-naïve individuals, a result that agreed with earlier studies reported worldwide. Hence, individuals with a history of SARS-CoV-2 infection showed a notably higher peak level of antibodies and a longer duration of the antibody presence for both the viral vector and RNA vaccines (ChAdOX1 and BNT162b2, respectively) [22,48,49,50]. Recent evidence suggested a single dose of mRNA vaccine in sero-positive, convalescent patients had elicited comparable antibody titers to sero-negative individuals who had received two doses of mRNA vaccine [51]. These observations illustrated the booster effect of vaccination against a background of previous infection by SARS-CoV-2 could induce a good IgA response [47].

It has been shown that vaccines against COVID-19 protected the vaccinees against symptomatic disease but not necessarily against the occurrence of infection [52]. During the follow-up of our cohort, 81 cases of breakthrough infections occurred in both groups. Given that the collection of the samples was between 2021 and 2022, it was likely that the breakthrough infections, especially in the SARS-CoV-2-experienced individuals, was due to the shift in antigenicity from the previously encountered variants (likely D614G or delta–omicron). Moreover, we observed a higher rate of breakthrough infections in the SARS-CoV-2-experienced group who had received RNA vaccines, despite the higher titers of antibodies developed by this group. It is important to note that breakthrough infections, including reinfections, can still occur among vaccinated individuals. These breakthrough cases are typically associated with milder symptoms, as compared to those in unvaccinated individuals. It is crucial to recognize that the primary goal of COVID-19 vaccines is to prevent severe disease, hospitalization, and death. While breakthrough infections may happen, the vaccines have remained effective, providing a substantial level of protection against severe outcomes [53]. Interestingly, in SARS-CoV-2-naïve individuals, the highest rate of breakthrough infections was noted in the alum-adjuvanted inactivated-vaccine group. Notably, in this group, the rate of breakthrough infections dropped significantly in the group with previous infection, suggesting that alum-adjuvanted inactivated vaccine were more effective in previously infected individuals.

Despite using different designs, all COVID-19 vaccines share a common goal, which is to trigger robust immunity with a strong antibody response associated with the neutralization of SARS-CoV-2. Although it has been well established that individuals develop heterogeneous immune responses and antibody levels following vaccination, age has been a pivotal factor influencing the antibody response [54,55,56]. Numerous studies investigating vaccination against influenza, hepatitis A, hepatitis B, pneumococcus, tick-borne encephalitis (TBE), tetanus, and SARS-CoV-2 have consistently shown that the antibody response following vaccination decreased with increasing age [33,57,58,59]. Indeed, T-cell-dependent antibody production and B-lymphocyte generation decreased with age [54]. Consequently, the antibody response against infectious agents and following vaccination might not be adequate for older individuals [60]. Similar results were shown in the case of SARS-CoV-2 infection [61,62,63]. However, our study detected a decline in antibody titers with age for different vaccine platforms, except in viral-vector SARS-CoV-2-experienced recipients, in whom we noted a moderate positive association between the IgG levels and age.

This study had some limitations. Indeed, the sample size in our study presented limitations to our investigation, in particular, at the later (T4 and T5) follow-up visits. In addition, as the study was conducted with a specific group of employees, the selection of the participants may not have reflected the general population since children were not included in our study. Herein, we only assessed the systemic IgA levels. Some reports [25,46] showed modest correlations between mucosal and systemic IgA levels induced by SARS-CoV-2 antibodies. It has been suggested that systemic IgA antibody level may mediate protection against breakthrough infection [25]. The current study did not evaluate responses elicited against variants. In fact, mutations in the S protein have led to the emergence of multiple variants, known as variants of concern (VOC), leading to diverse phenotypes that have affected transmission and the sensitivity to antibody neutralization. Although spike mutations reduced vaccine efficacy due to the decreased neutralizing activity [64,65,66,67,68,69,70], it is important to note that the Fc-dependent effector functions could still play a role in reducing the incidence of severe disease following vaccination [71]. Additionally, we were not able to assess the specific neutralizing antibodies to SARS-CoV-2 in the collected sera, which could have added meaningful information for the post-vaccination responses. However, post-SARS-CoV-2 vaccination, data have supported the correlation between the neutralizing antibodies and the anti-S-RBD antibodies [72,73,74,75]. Finally, we did not evaluate factors other than age that could influence the immune response following vaccination, such as smoking and obesity [76].

## 5. Conclusions

Overall, our data, obtained from a sample group from the Tunisian population, showed that COVID-19 RNA vaccines induced the highest levels of IgA and IgG specific antibodies, as compared to alum-adjuvanted inactivated and viral vector vaccines, in SARS-CoV-2-naïve or previously infected individuals. In addition, our data confirmed that individuals who had an earlier COVID-19 infection and received a vaccination developed a more robust antibody response, as compared to those who had been vaccinated without prior infection. Finally, these individuals exhibited a higher level of IgA antibody response.

## Figures and Tables

**Table 1:** Vaccinations received by SARS-CoV-2-naïve and SARS-CoV-2-experienced individuals.

	SARS-CoV-2-Naïve at Pre-Vaccination (*n* = 93)			SARS-CoV-2-Experienced at Pre-Vaccination (*n* = 93)		
	Inactivated Vaccines	RNA Vaccines	Viral Vector Vaccines	Inactivated Vaccines	RNA Vaccines	Viral Vector Vaccines
Donors, N	15	65	13	7	68	18
N, (Sex)	8 (F) 7 (M)	38 (F) 27 (M)	7 (F) 6 (M)	4 (F) 3 (M)	46 (F) 22 (M)	11 (F) 7 (M)
Age, median (range)	49 (29–62)	44 (25–71)	46 (30–67)	41.5 (34–62)	45 (26–70)	46 (23–67)
Boosted, N (Time-point)	4 (T3) 2 (T4)	6 (T3) 27 (T4) 2 (T5)	1 (T3) 3 (T4)	3 (T3) 1 (T4)	11 (T3) 22 (T4) 1 (T5)	1 (T3)
Booster Categories, N (category)	1 * 5 **	34 * 1 **	4 **	4 **	34 *	1 *
Boosted total	45/93			39/93		

*: Homologous booster vaccine; **: Heterologous booster vaccine.

**Table 2:** Breakthrough infections upon SARS-CoV-2 vaccination.

	SARS-CoV-2-Naïve at Pre-Vaccination (*n* = 93)			SARS-CoV-2-Experienced at Pre-Vaccination (*n* = 93)		
	Inactivated Vaccines	RNA Vaccines	Viral Vector Vaccines	Inactivated Vaccines	RNA Vaccines	Viral Vector Vaccines
Donors, N	15	65	13	7	68	18
Breakthrough infection, N (Time-point)	5 (T3) 4 (T4) 1 (T5)	5 (T2) 1 (T3) 6 (T4) 19 (T5)	5 (T3)	1 (T4)	4 (T3) 13 (T4) 14 (T5)	2 (T2) 1 (T3)
Breakthrough infection, N (%)	10 (66%)	31 (47%)	5 (38%)	1 (14%)	31 (45%)	3 (18%)
Breakthrough infection total	46/93			35/93		

## Data Availability

Data are contained within the article and Supplementary Materials.

## Supplementary Materials

The following supporting information can be downloaded at: https://www.mdpi.com/article/10.3390/tropicalmed9030061/s1, Figure S1: S-RBD-based ELISA assay validation; Figure S2: Correlation between age with IgG antibody levels following viral vector vaccination.
