# Supplementary material for: A Longitudinal Study in Tunisia to Assess the Anti-RBD IgG and IgA Responses Induced by Three Different COVID-19 Vaccine Platforms
Source: Trop Med Infect Dis. 2024 Mar 13;9(3):61. doi: 10.3390/tropicalmed9030061 (PMC10975151; doi:10.3390/tropicalmed9030061)
Supplement: Supplementary file 1 [file tropicalmed-09-00061-s001.zip › tropicalmed-2697102-supplementary.pdf]

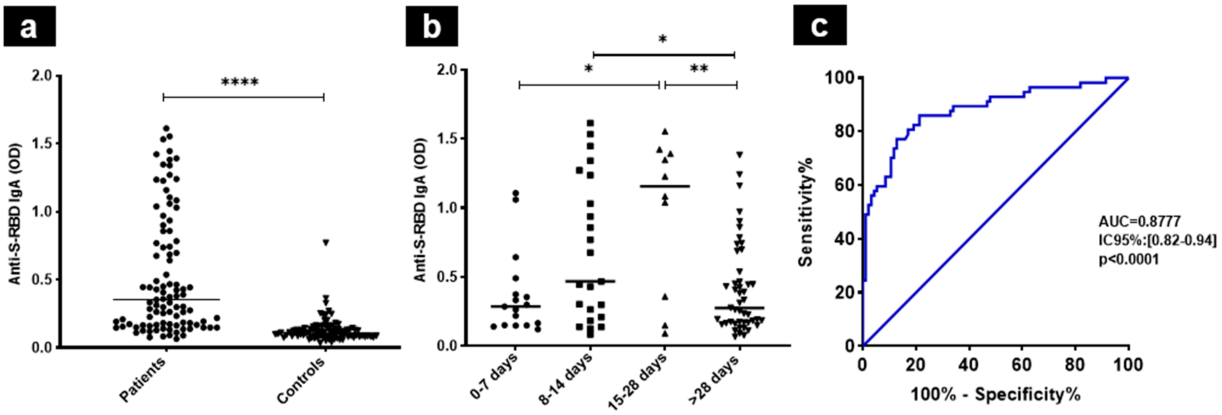

Figure S1. S-RBD-based ELISA assay validation. A total of 110 sera/plasma samples obtained from COVID-19 patients were confirmed by PCR (collected within the first six weeks after the onset of illness symptoms), and 72 sera samples collected before the pandemic (before 2018) from pre-pandemic sera were subjected to antibody detection. **(a)** COVID-19 sera exhibited significantly higher ODs than pre-pandemic control sera in both tests ($P < 0.0001$). S-RBD specific IgA levels are presented as optical densities (OD450nm–620nm); **(b)** S-RBD specific IgA levels obtained in COVID-19 patients were stratified according to the blood collection time after the onset of the symptoms. The highest IgA levels were obtained with sera collected between day 15 and day 28; **(c)** ROC curve analysis representing the performance of the S-RBD-based ELISA test performance was estimated by the area under the ROC curve (AUC) established using the RT-PCR test as a gold standard. The overall performance of the anti-S-RBD ELISA was high (AUC=0.877, $p < 0.0001$) with a sensitivity of 83% and a specificity of 95%. Bars indicate median values. Mann-Whitney t-test was used to compare differences between the two groups, a 2-tailed P-value <0.05 was considered statistically significant. *** $P \leq 0.001$, ** $P \leq 0.01$ and *$P \leq 0.05$

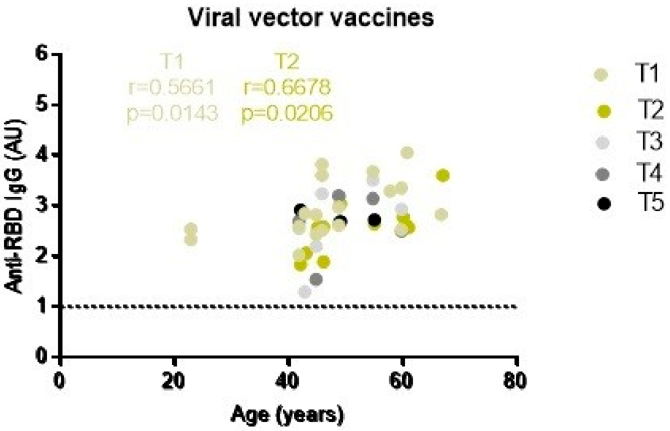

Figure S2. Correlation between age with IgG antibody levels following viral vector vaccination. The graph shows IgG antibody against RBD (AU, Y-axis) plotted against age (years, X-axis) 3-4 weeks after the first immunization (T1, light yellow), 3 months (T2, dark yellow), 5 months (T3, light grey), 7 months (T4, dark grey), and 12 months after that (T5, black). Associations were calculated using a nonparametric Spearman correlation with a 95% confidence interval and a two-tailed p-value <0.05 was statistically significant
